# Diagnosis of Clostridioides difficile infection and impact of testing

**DOI:** 10.1099/jmm.0.001939

**Published:** 2024-12-03

**Authors:** Virginie F. Viprey, Emma Clark, Kerrie A. Davies

**Affiliations:** 1 Healthcare Associated Infections Research Group, Leeds Institute of Medical Research, University of Leeds, Leeds, UK; 2 National Institute for Health and Care Research (NIHR), Leeds Biomedical Research Centre (BRC), Leeds, UK; 3 Department of Microbiology, Leeds Teaching Hospitals NHS Trust, Leeds, UK

**Keywords:** *Clostridioides difficile *infection, CDI, laboratory diagnosis

## Abstract

Diagnosis of *Clostridioides difficile* infection (CDI) remains challenging as it involves in the first instance recognition (clinical awareness) of the patients’ symptoms for clinical suspicion of CDI to warrant testing, and secondly, different laboratory tests have been described for CDI. Due to the overwhelming amount of information in the literature on CDI tests and their performance, with separately published guidelines, this review aims to provide a comprehensive but concise summary of the current state of CDI diagnostic testing. Current knowledge and the impact of using different laboratory diagnostic procedures for CDI, including the most recommended approach as a two-step algorithm and the concept of diagnostic stewardship, are being discussed. This review provides an updated overview and valuable take-home messages in the field of CDI laboratory testing and highlights that timely diagnosis is important for the clinical management of CDI and that the recommended testing procedures are increasingly becoming more widely accepted.

## Introduction

*Clostridioides difficile* infection (CDI) has been identified as one of the leading causes of nosocomial infection and antibiotic-associated diarrhoea worldwide and of increasing concern in the community [1,3]. CDI is associated with significant morbidity, mortality and profound economic burden [4,7]. Timely, accurate diagnosis and prevention of CDI is therefore imperative.

*C. difficile* is an anaerobic spore-forming bacillus that exists ubiquitously in the environment and can be carried asymptomatically within the gastrointestinal (GI) tract of healthy individuals. The phenomenon known as colonization resistance enables *C. difficile* to be present in the gut without causing any clinical signs or symptoms [8]. However, perturbation of the healthy host gut microbiome, for example, by antibiotic challenge, provides a niche for the outgrowth and proliferation of *C. difficile* as a pathogen, thus inducing CDI [9].

In the majority of cases, clinical manifestations of CDI range from mild-to-severe diarrhoea, with or without abdominal cramping and colitis (including pseudomembraneous colitis) with an overall mortality rate of ~17% [10]. In severe cases, patients may develop paralytic ileus or toxic megacolon accompanied by symptoms of pain, fever, vomiting, tachycardia and lethargy [11]. A recent commentary on patient involvement in CDI research highlighted the long-term impact on quality of life, with patients reporting feelings of fear about getting recurrent disease [12].

The organism has a cell surface enzyme, glutamate dehydrogenase (GDH), which can be used as a diagnostic marker for *C. difficile*. Some *C. difficile* strain types are toxigenic, carrying toxin genes that enable the organism to produce both toxin A (*TcdA*) and toxin B (*TcdB*), which act synergistically and mediate the pathogenicity of the organism [13]. *C. difficile* toxins cause inflammation of the gut due to their disruptive effect on the actin skeleton and epithelial barrier integrity, leading to cell death and tissue damage [13,15]. Detection of toxin production and of their cytotoxic effect (rounding) on cells can therefore be exploited for diagnosis.

*C. difficile* diagnostics must differentiate between colonization and infection in order to establish the disease status of the patient [16]. This is essential so that the correct treatment and infection prevention and control (IPC) measures can be implemented to prevent transmission of the organism and reduce morbidity and mortality associated with the disease [17]. Laboratory diagnosis of CDI is however a complex and contentious subject. Recommendations between guidelines vary, with comparison studies complicated by the fact that there are two reference methods, cytotoxigenic culture and cell cytotoxicity neutralization assays (CCNAs), which detect slightly different things, that is, the presence of a toxigenic strain (not necessarily infection) and infection (detection of toxin production in the faecal sample), respectively [18]. The decision of which diagnostic assay to use is further compounded by the issue of what to detect, the organism, its toxins, or its DNA [19].

This narrative review aims to provide up-to-date information on the impact of using different diagnostic tools for CDI, alongside recommended procedure guidelines. While clinical parameters and target population for testing are outside the scope of this review, it should be noted that the criteria for a sample to be tested for CDI is that it should be a loose stool specimen and collected from patients >2 years of age with three or more unexplained unformed stools in 24 h [20].

Specifically, this review focuses on the importance of distinguishing between laboratory tests that can be used to detect only the presence of *C. difficile* (i.e. the organism or its DNA, regardless of toxin production status in the original faecal sample) and those that detect toxin production in the original sample (sections on ‘Detection of *C. difficile*’ and ‘Detection of *in vivo* toxin production’, respectively). Recommended approaches to diagnosis, as an algorithm using those laboratory tests in combination, are presented in the section on ‘Recommended testing methods’. New technologies and assays are discussed in sections on ‘New technologies’ and ‘Additional laboratory assays to facilitate diagnosis’. The impact of testing with a focus on diagnostic stewardship is summarized in the section on ‘Impact of testing’.

## Laboratory diagnosis of *C. difficile* infection

### Detection of *C. difficile*

Detection of *C. difficile* using GDH enzyme immunoassay (EIAs) indicates the presence of the organism but does not distinguish between toxigenic and non-toxigenic *C. difficile*. A meta-analysis published in 2023 described the sensitivity and specificity of GDH EIAs as 82% [95% confidence interval (CI): 79–84%] and 91% (95% CI: 90–92%), respectively [21].

Nucleic acid amplification tests (NAATs) are used for the detection of toxin gene DNA and can offer high-throughput and high-sensitivity solutions for the detection of toxigenic *C. difficile*. NAAT may also be more sensitive than GDH assays for the detection of certain PCR ribotypes of *C. difficile*, although this has only been demonstrated in one small study [22]. Several commercial *C. difficile* NAATs are available on the market, mostly PCR assays, and have all been described to achieve similar high sensitivity (>92%) and specificity (>99%) [21,23, 24]. However, the main limitation of NAAT alone for the diagnosis of *C. difficile* infection is that a positive NAAT result indicates the presence of the toxin gene only, and therefore, the presence of a toxigenic strain in faecal samples, but does not confirm infection (caused by production of toxin molecules). NAAT does not therefore differentiate between colonization by a toxigenic strain (presence only of *C. difficile*) and infection by a toxigenic strain (*C. difficile* that produces toxin, leading to disease). While most NAATs on the market are PCR-based technology, some also utilize loop-mediated isothermal amplification (LAMP), a rapid and cost-effective tool for the diagnosis of infectious disease. The same caveats apply for LAMP, as for PCR-based NAATs, they rely on the detection of the toxin gene and therefore cannot differentiate between colonization only and the disease [25,28]. It is worth noting that some NAATs have been developed to identify a third so-called binary toxin gene, as some *C. difficile* strains causing CDI were reported to be toxin A/B negative but binary toxin positive [23,29], although the role of binary toxin in pathogenesis is unclear.

The use of NAAT alone for diagnosis can result in overdiagnosis of *C. difficile* and unnecessary treatment in patients who do not have an infection [30]. Indeed, CDI-related mortality rate was found to be significantly lower in patients who had a NAAT-positive but toxin-negative test results compared to those who were both NAAT and toxin positive (0.6% vs. 8.4%, *P*=0.001) [30]. A negative NAAT can however rule out the presence of *C. difficile* and can therefore be useful for screening patients who present with GI symptoms [31]. The use of quantification methodology applied to NAAT (cycle threshold values) has been found to be able to predict toxin status and poor outcomes with some degree of accuracy (~80%) but should not however replace testing for the presence of toxin [32].

Similarly, cytotoxigenic culture assays (CT) cannot differentiate colonization and disease, as they detect *C. difficile* strains from a sample, with the ability to produce toxin when cultured in a laboratory, but do not confirm the presence of toxins in the original faecal sample. As discussed earlier, the detection of toxin in the original sample, not the detection of an isolate that has the ability to produce toxin in a laboratory setting, has been correlated with increased mortality [19]. Furthermore, the long turn-around time and expertise required to carry out a CT assay limit its use as a diagnostic tool; it is however often used as a reference assay for comparison studies.

### Detection of *in vivo* toxin production

CCNA is the most specific test for CDI and is the recognized gold standard for the detection of toxin molecules produced by *C. difficile* in faecal samples [19,21]. This technique provides a visual representation of the effect of the cytotoxin on cell monolayers but can be time-consuming and subjective, requiring specialized expertise for interpretation. Standardization of CCNA protocols is also problematic due to the lack of agreement across studies on the faecal sample dilutions and cell lines to use [31]. While semi-automated CCNAs have been described to reduce the risk of human errors that can be encountered with manual CCNA, this is still time-consuming and not a reasonable practical solution for clinical testing [33].

EIAs (coloured result in a microtitre well) and immunochromatography tests (visible line on a substrate strip) were developed to overcome the time limitations of CCNA. The variable performance of different commercially available EIAs was demonstrated as far back as 2008, while a recent meta-analysis reported the sensitivity of toxin EIAs at 75% and specificity at 95% [21,34, 35]. While the first EIAs were developed to identify toxin A only [36], most EIAs can now detect both toxins A and B, which is of importance as toxin A-negative, but toxin B-positive strains have been found to cause disease [37,38].

### Recommended testing methods

Diagnosis of CDI should not be based only on toxin detection by EIA or be based solely on results from molecular assays. Inappropriate positive predictive values have been described at low disease prevalence when using a single test, meaning that a high percentage of patients with a positive test for toxin EIAs or NAAT (~19% if using toxin EIAs alone or ~54% if using NAAT alone) may not have the disease [31].

To overcome the limitations of all the available tests, lack of sensitivity for toxin EIAs and lack of specificity for CDI for the GDH EIAs and NAATs, algorithms combining tests are now recommended. The European Society of Clinical Microbiology and Infectious Diseases (ESCMID) guidelines recommend detection of the organism first using either GDH EIAs or NAAT, followed by toxin detection by EIA [31]. The 2016 ESCMID diagnostic guidance document described in detail the source of information used to develop those guidelines, one of which being the 2012 UK National Health Service recommendations for a combined testing algorithm [39]. According to those published European guidelines, the choice of which first test to use (GDH EIAS or NAAT) can be decided by each individual institution [31]. For instance, a nationwide study describing switching to NAAT as a first step instead of GDH EIA confirmed that this did not lead to increased CDI rates [40]. For the second step, the use of poorly performing (low sensitivity) toxin EIAs will however still reduce the overall algorithm sensitivity; it is, therefore, essential to select the best-performing toxin EIA available. The use of two-step sequential algorithms is also recommended by the European Centre for Disease Prevention and Control (ECDC), the Infectious Diseases Society of America (IDSA) and the Society for Healthcare Epidemiology of America (SHEA) guidelines [20,41]. Commercial assays have been developed to allow simultaneous detection of GDH and toxin A/B, providing a two-step algorithm solution in one test; the result should still be interpreted sequentially, however.

Additional optional steps for CDI diagnosis are recommended in cases that present with a positive GDH test result but a toxin A/B EIA-negative test result [20,31, 41], such as including NAAT or cytotoxigenic culture test. This can provide additional information on the presence of a toxigenic strain, useful for IPC considerations. It is worth noting that the IDSA guidelines also consider that the use of NAAT alone is acceptable if used alongside clinical criteria [20]. This could be problematic as a switch to NAAT alone may cause overdiagnosis of CDI and has indeed been found associated with a 56% increase in the healthcare-associated CDI rate [42]. Interestingly, a recent systematic review and meta-analysis found that patients with a NAAT-positive but toxin EIA-negative test result may benefit from treatment, but these results could be impacted by the use of poor-quality toxin tests that may miss some true-positive cases [43]. This possible underreporting of CDI cases that were NAAT positive but toxin EIA negative was raised by another group, highlighting the potential issue of management of patients with toxin-negative status by conventional EIAs, with laboratory interpretative comments to the test report found to influence the decision to treat for CDI [44]. This is a reminder that the diagnosis of CDI should be based on the clinical presentation of the patient, assisted by laboratory diagnostics, not based on laboratory diagnostics alone. Clinical assessment is essential in light of a NAAT-positive but toxin-negative result, especially where a toxin test with poor sensitivity has been used.

### New technologies

Ultrasensitive assays for the detection of *C. difficile* toxins have been developed; however, although achieving higher sensitivity than toxin EIA, those assays are not currently available for commercial applications [45,47]. This is disappointing, and further development of more accurate toxin detection assays, especially those that could be used near-patient, is warranted.

*C. difficile* has been included as a target within several molecular GI panels. However, similarly to stand-alone NAAT, these assays detect the presence of toxigenic *C. difficile* rather than the production of toxin in the original sample, meaning that they are not recommended as a stand-alone assay for the diagnosis of CDI. Moreover, the inclusion of *C. difficile* in multiplex GI panels was found to lead to CDI overdiagnosis, increasing costs associated with unnecessary CDI test ordering and treatment and potentially delaying the diagnosis of alternative causes of diarrhoea [48]. A recent retrospective multicentre study demonstrated that most patients with a positive *C. difficile* test result from a GI panel (91%, *n*=444/489) were reflexed to further CDI testing, with only 24% of those patients confirmed as CDI by the two-step algorithm (GDH/toxin EIAs) [48]. Similarly, in another study describing the use of multiplex GI panels for screening haemopoietic stem cell transplant patients, confirmation of toxin positivity status was only found in 38% of patients that had a positive *C. difficile* multiplex GI panel result [49].

POC molecular assays (NAAT-based) for the detection of the *C. difficile* toxin gene have been developed, with studies reporting sensitivity of 85–97% and specificity of 97–99% [50,51]. This would move testing for CDI closer to the patients in a clinical setting, with further confirmatory laboratory testing applied (toxin EIAs) on those who are positive for the NAAT. A cost–consequence model study found that such a strategy was likely to translate into cost savings for hospitals that do not have a testing laboratory onsite; cost savings were mainly attributed to the faster turnaround time of POC tests [52]. Future research is, however, needed to establish the full clinical performance of such POC tests compared to other NAAT-based assays, and whether the introduction of POC testing results in more effective patient management.

### Additional laboratory assays to facilitate diagnosis

Laboratory tests that reflect the intestinal inflammation status of patients can assist the diagnosis of patients with severe disease and therefore clinical management [53].

Both faecal calprotectin (fCP) and lactoferrin are proteins derived from polymorphonuclear leucocytes, which are released in the gastrointestinal tract at high concentrations in response to mucosal inflammation and infections and have been investigated as biomarkers of gut disease [54,55].

High levels of lactoferrin have been shown to be associated with CDI severity and may be able to predict recurrence [56,57]. Several studies have been conducted to evaluate the utility of fCP for assessing the severity of CDI. Elevated fCPs were found in patients with CDI compared to healthy controls and in severe CDI compared to milder cases [53]. Furthermore, the measurement of fCP was found potentially useful in identifying patients with CDI who would benefit from multiple faecal microbiota transplantation infusions [58]. There is also evidence demonstrating that fCP levels are higher in patients with CDI versus patients with diarrhoea of other aetiologies, indicating that fCP may be useful for screening patients with diarrhoea for CDI [59,61]. Higher levels of fCP have also been observed in patients with CDI versus those with asymptomatic colonization, but the difference was statistically insignificant [62]. fCP may be elevated in proportion to the degree of inflammation caused by CDI, thus highlighting its potential to indicate the severity of the disease [63]. However, larger prospective studies are required to provide sufficient evidence to determine the utility of fCP as a diagnostic marker [63]. Swale *et al.* [61] found a significant correlation between raised lactoferrin and severity of CDI, whereas fCP was raised, but not significantly [61]. Lactoferrin has been suggested as an adjunct to a test algorithm for CDI [64].

Both biomarkers have limited clinical applicability and only serve as an adjunct to the current diagnostic algorithms in place.

## Impact of testing

Timely diagnosis of CDI is important for patients but is also necessary for the control of outbreaks; CDI has a long-lasting impact on the quality of life, and therefore, early diagnosis and management of patients with the disease will contribute to reducing the burden of CDI [7].

Selection of patients who should be tested for CDI is a key concept of diagnostic stewardship, which is aimed at reducing inappropriate testing, false positives and unnecessary antibiotic prescriptions [65]. Diagnostic stewardship is encouraged and endorsed by the IDSA as part of the US diagnostic guideline recommendations [66,67].

Interventions (strict criteria and education) to reduce unnecessary NAATs have been described in several studies and are associated with a reduction in the detection of false positives due to laxative use, reported CDI rates and healthcare costs [68,70].

Electronic clinical decision support tools provide diagnostic guidance at the POC and have been found to impact *C. difficile* testing in a recent systematic review, namely a reduction in CDI testing and rates [71]. Implementations of diagnostic stewardship interventions that included a clinical decision support tool and restriction on NAAT reflex testing have been found associated with a 33% reduction in *C. difficile* test order and a 57% reduction in reported hospital-onset CDI [72]. The main reported drawback of electronic clinical decision tools, however, is the high number of alerts sent out, leading to the so-called alert fatigue and clinical override [71,73]. For instance, a study assessing the impact of an alert to notify that patient received laxative within 24 h prior to CDI testing found that 75% of those were overridden and tests ordered regardless [74]. On the other hand, intervention with a stringent approval process for test ordering was found successful in reducing unnecessary testing for *C. difficile* [75].

While diagnostic stewardship has many benefits, one of the possible drawbacks or concerns is the possibility that this could lead to underdiagnosis. For example, low clinical suspicion for CDI in the community has been reported, leading to underdiagnosis in this setting [76]. Low testing rates in some countries have also been linked to underdiagnosis [77]. Early detection of CDI in the community may be hampered by reliance on risk factors such as older age and previous antibiotic use to select patients for testing. However, such selective testing can lead to missed cases. For instance, a recent point-prevalence study across Europe found that half of community CDI cases had no previous history of antibiotic use and that almost all undiagnosed adult CDI community cases were younger than 65 years old [76]. While low levels of testing may lead to underdiagnosis of CDI, overdiagnosis is also problematic and associated with increased costs [78,79]. A study conducted in European hospitals in 2015 found that the highest number of CDI cases were reported in hospitals that use a stand-alone test, or algorithm, without detection of toxin (GDH/NAAT or NAAT), suggesting that overdiagnosis of CDI occurs when non-recommended testing strategies are used to diagnose CDI [80]. It is, however, encouraging that the recommended diagnostic tests for CDI were reported to be used by over 80% of hospitals in a recent European survey (2018–2019) compared with just over half a decade ago [81].

Transition to two-step testing (NAAT/toxin) raised furthermore the issue of management of patients with a NAAT-positive test but a toxin EIA-negative test result (indicative of colonization only). A study in the USA comparing CDI treatment rates in the periods pre- and post-introduction of two-step testing found a reduction in CDI-related antibiotic prescription in the two-step testing era, whereby 41% of patients colonized (NAAT positive and toxin negative) were treated compared to 93% of patients who were NAAT positive in the single test era [82]. The introduction of two-step methodologies for CDI testing is therefore important for antimicrobial stewardship, leading to the reduction of unnecessary antibiotic prescriptions in some colonized patients. Some studies have highlighted that withdrawal of treatment for NAAT-positive/toxin-negative cases had no adverse outcome, while others found that a small subset of those cases had the disease, highlighting that decision on whether to treat should also be based on clinical parameters and risk factors [44,83,85].

## Conclusion

Clinical findings combined with diagnostic stewardship and the use of a recommended two-step algorithmic approach for testing can optimize the accurate diagnosis of CDI, thus informing appropriate patient management and clinical outcomes. Encouragingly, recommended approaches to improve CDI diagnosis are being more widely considered.

## Abbreviations

CCNAs: cell cytotoxicity neutralization assays
CDI: *Clostridioides difficile* infection
CI: confidence interval
ECDC: European Centre for Disease Prevention and Control
EIAs: enzyme immunoassays
ESCMID: European Society of Clinical Microbiology and Infectious Diseases
fCP: faecal calprotectin
GDH: glutamate dehydrogenase
IDSA: Infectious Diseases Society of America
IPC: infection prevention and control
LAMP: loop-mediated isothermal amplification
NAATs: Nucleic acid amplification tests
SHEA: Society for Healthcare Epidemiology of America
TcdA: toxin A
TcdB: toxin B
